# HIV-Associated TB in An Giang Province, Vietnam, 2001–2004: Epidemiology and TB Treatment Outcomes

**DOI:** 10.1371/journal.pone.0000507

**Published:** 2007-06-06

**Authors:** Trinh Thanh Thuy, N. Sarita Shah, Mai Hoang Anh, Do Trong Nghia, Duong Thom, Truong Linh, Dinh Ngoc Sy, Bui Duc Duong, Luu Thi Minh Chau, Phung Thi Phuong Mai, Charles D. Wells, Kayla F. Laserson, Jay K. Varma

**Affiliations:** 1 Global AIDS Program, U.S. Centers for Disease Control and Prevention, Hanoi, Vietnam; 2 Division of Tuberculosis Elimination, U.S. Centers for Disease Control and Prevention, Atlanta, Georgia, United States of America; 3 An Giang Province Preventive Medical Center, Long Xuyen City, Vietnam; 4 National Hospital of Tuberculosis and Lung Diseases, Vietnam National TB Program, Ministry of Health, Hanoi, Vietnam; 5 LIFE-GAP Office, Ministry of Health, Hanoi, Vietnam; 6 Thailand Ministry of Public Health – U.S. Centers for Disease Control Collaboration, Bangkok, Thailand; Medical University of South Carolina, United States of America

## Abstract

**Background:**

Mortality is high in HIV-infected TB patients, but few studies from Southeast Asia have documented the benefits of interventions, such as co-trimoxazole (CTX), in reducing mortality during TB treatment. To help guide policy in Vietnam, we studied the epidemiology of HIV-associated TB in one province and examined factors associated with outcomes, including the impact of CTX use.

**Methodology/Principal Findings:**

We retrospectively abstracted data for all HIV-infected persons diagnosed with TB from 2001–2004 in An Giang, a province in southern Vietnam in which TB patients receive HIV counseling and testing. We used standard WHO definitions to classify TB treatment outcomes. We conducted multivariate analysis to identify risk factors for the composite outcome of death, default, or treatment failure during TB treatment. From 2001–2004, 637 HIV-infected TB patients were diagnosed in An Giang. Of these, 501 (79%) were male, 321 (50%) were aged 25–34 years, and the most common self-reported HIV risk factor was sex with a commercial sex worker in 221 (35%). TB was classified as smear-positive in 531 (83%). During TB treatment, 167 (26%) patients died, 9 (1%) defaulted, and 6 (1%) failed treatment. Of 454 patients who took CTX, 116 (26%) had an unsuccessful outcome compared with 33 (70%) of 47 patients who did not take CTX (relative risk, 0.4; 95% confidence interval [CI], 0.3–0.5). Adjusting for male sex, rural residence, TB smear status and disease location, and the occurrence of adverse events during TB treatment in multivariate analysis, the benefit of CTX persisted (adjusted odds ratio for unsuccessful outcome 0.1; CI, 0.1–0.3).

**Conclusions/Significance:**

In An Giang, Vietnam, HIV-associated TB was associated with poor TB treatment outcomes. Outcomes were significantly better in those taking CTX. This finding suggests that Vietnam should consider applying WHO recommendations to prescribe CTX to all HIV-infected TB patients.

## Introduction

HIV has contributed to a global resurgence of tuberculosis (TB).[1] Despite successful implementation of World Health Organization (WHO)-recommended TB control strategies, many high-burden TB countries have seen stable or rising TB case notifications due to HIV-associated TB.[2] HIV fuels the TB epidemic in several ways. HIV-infected persons have a 50% lifetime risk of progressing from latent TB infection to TB disease, compared with 5–10% of HIV-uninfected persons.[1] HIV-infected persons are more likely than HIV-uninfected persons to have acid-fast bacilli (AFB) smear-negative sputum results, with up to 60% having negative sputum smears.[3] This may delay diagnosis and treatment of TB, potentially leading to greater TB transmission and mortality. Compared with HIV-uninfected TB patients, HIV-infected TB patients have substantially higher case fatality rates, which can be reduced through co-trimoxazole (CTX) and anti-retroviral therapy (ART).[4]–[7]

Vietnam ranks 13^th^ among the 22 countries designated by WHO as having the world's highest TB burden.[2] Vietnam was one of the first of these countries to reach WHO targets for successful DOTS implementation, including >70% case detection and >85% cure for new, smear-positive TB cases. Nevertheless, case notifications have been rising in Vietnam, and HIV is likely an important cause.[2] Vietnam's HIV epidemic is concentrated in urban areas of several provinces and high-risk populations, e.g. injection drug users, commercial sex workers, and clients of commercial sex workers. Overall, UNAIDS estimates that 260,000 HIV-infected people were living in Vietnam in 2005, including an estimated 0.5% of the population aged 15–49 years.[8] In 2005, HIV-associated TB accounted for approximately 4.3% of all TB cases nationally and as many as 9.8% of cases in Ho Chi Minh City in 2005. (Vietnam Ministry of Health, unpublished)

In Vietnam, HIV-infected TB patients have a high mortality rate, but limited information is known about risk factors for death. One study from Ho Chi Minh City examined the relationship between HIV, anti-TB drug-resistance, and TB treatment outcomes in 2196 TB patients registered from 1998–2000.[9] This study found that the mortality rate for HIV-infected, smear-positive TB patients was 34%, compared with 3% in HIV-uninfected patients. This study only examined outcomes for a small number of HIV patients (44 total) and did not analyze factors associated with poor treatment outcomes in HIV-infected TB patients.

An Giang province (2005 population 2,194,218) has the highest provincial TB notification rate in Vietnam (252 cases per 100,000 in 2004), and the fifth highest HIV prevalence (0.6%).(Vietnam Ministry of Health, unpublished) Unlike most provinces in Vietnam, TB patients in An Giang routinely receive HIV counseling and testing and HIV patients are screened regularly by chest radiography for TB.(Vietnam Ministry of Health, unpublished) In 2004, HIV prevalence in TB patients was 4.8%. We conducted an evaluation of TB and HIV collaboration in An Giang province to help guide policies and strategies for scaling up TB/HIV collaboration in Vietnam. As part of this larger evaluation, we conducted a retrospective cohort study of all HIV-infected TB patients diagnosed in An Giang province from 2001–2004 in order to improve our understanding of the local epidemiology and to identify factors associated with TB treatment outcomes.

## Methods

### Diagnosis of TB and HIV in An Giang

In An Giang, persons suspected of having TB are referred to TB clinics at district health centers or to the TB clinic at the provincial hospital for a standardized evaluation, including history and physical examination, examination of three sputum specimens for AFB, and, if AFB smear-negative, chest radiography. TB diagnosis and classification follow standard WHO definitions.[10] All persons diagnosed with TB in these facilities undergo HIV counseling and testing. A rapid serological test is performed in the TB clinic and, if positive, is sent to a central laboratory at the province's Preventive Medicine Center [PMC] for repeat HIV serology. HIV testing in the laboratory is by enzyme-linked immunosorbent assay (Genscreen, Sanofi, Paris, France or Serodia, Fujirebio, Tokyo, Japan) followed by two different HIV ELISA tests if the first is positive. The tests used varied over the time of this project.

From 2001 – 2004, HIV-infected patients enrolled in An Giang's home-based care program were also routinely referred for chest radiography to screen for pulmonary TB, regardless of symptoms. In 2004, 61% of 357 HIV-infected persons enrolled in home-based care received at least one chest radiograph. Patients with abnormal chest radiographs were referred for further evaluation at a TB clinic, but 90% did not follow-up with such referrals.(CDC, unpublished)

In addition to routine clinical records and standardized TB registers, TB clinics also maintain a separate register of HIV-infected TB patients. District health centers and An Giang province maintain clinical records and a register of persons diagnosed with HIV.

### Data Collection

We retrospectively reviewed TB, HIV, and TB/HIV registers and clinical records to collect data about all HIV-infected TB patients aged ≥15 years diagnosed in An Giang province from 2001 to 2004. Data were abstracted from these records using a standardized form that included questions about personal and demographic history; HIV risk factors, conditions, and treatment; TB diagnostic studies, treatment history, and treatment outcomes. Public health staff were trained to complete the abstraction form, and forms were checked for completeness and accuracy by a data collection supervisor.

### Definitions for Data Analysis

We used standard WHO definitions for TB disease classification, registration and treatment outcome categories. TB registration status was divided into “new,” “re-treatment,” or “transfer in.” The “re-treatment” group included cases that were classified as relapse, treatment after failure, or treatment after default. Final TB treatment outcome was divided into “successful” or “unsuccessful.” A “successful” treatment outcome included cured or completed; “unsuccessful” included failure, death, or default; three cases with an outcome of transfer out were excluded.

We divided age into five categories: 15–24 years, 25–34 years, 35–44 years, 45–54 years, and greater than 55 years. We divided districts into “urban” (Long Xuyen city and Chau Doc district) or “rural” (remaining nine districts).

TB clinical records indicated whether or not the patient suffered an adverse event during TB treatment. Clinical staff did not use a standardized definition for recording adverse events, and there was no supplemental documentation for what adverse event occurred. Staff reported that this category was most commonly used to indicate rash or intolerance to anti-TB medications.

HIV/AIDS clinical records collected data about occupation, education, religion, ethnicity, marital status, and HIV risk group using pre-specified options. We used these data elements and response options when collecting data. For analysis, we re-classified occupation as “employed” or “unemployed.” Persons were considered “employed” if they held any type of job, including: farmer, driver, worker (of any type), teacher, soldier/police, public servant, or unskilled laborer. Persons were considered “unemployed” if they had no job, were a housewife, or were only intermittently employed. Education was classified as secondary or below (including illiterate persons) vs. high school or above (including college or university). Marital status was classified as married, single, divorced, separated, and widowed.

HIV risk assessment categories that were pre-defined on public health forms included: men who have sex with men (MSM), sex with a commercial sex worker (CSW), sex with a stable partner, sex with multiple partners, heterosexual sex with spouse, injection drug user (IDU), HIV-infected mother (i.e., mother-to-child transmission) or other.

HIV/AIDS clinical records included whether a person had conditions consistent with HIV at the time of HIV diagnosis, including weight loss ≥10%, fever≥1 month, diarrhea≥1 month, TB, Candida esophagitis, neurological disorder, recurrent pneumonia, invasive cervical cancer, cryptococcal meningitis, Kaposi's sarcoma, generalized herpes infection, or other. We divided data about the presence of HIV symptoms into “yes” if any HIV symptom was noted (including other write-in symptoms) or “no.” No hematology or immunology laboratory data were available.

HIV/AIDS clinical records also indicated whether the patient was prescribed co-trimoxazole (CTX). Records did not indicate the dosage, frequency or duration of CTX and did not indicate whether the patient actually ingested the drug. An Giang HIV/AIDS program policy is to provide CTX to all HIV-infected persons free of charge. Current national policy is to provide CTX to HIV-infected persons with WHO stage 3 or 4, WHO stage 1 or 2 with CD4+ T-lymphocyte<200 cells/mm^3^, or WHO stage 2 with total lymphocyte count<1200 cells/mm^3^. CTX is not routinely provided in TB clinics. ART was not available in An Giang province during the years covered by this evaluation.

### Statistical Analysis

To describe patient characteristics, we calculated proportions and medians. For categorical variables, we compared proportions using chi-square tests and, when appropriate, Fisher's exact test. For continuous variables, we compared medians using the Wilcoxon Rank-Sum Test and evaluated trends using chi-squared test for trend. Rates were calculated using census data provided by local government authorities.

We compared HIV-infected TB patients with a successful TB treatment outcome to those with an unsuccessful outcome. In bivariate analysis, we analyzed risk factors for an unsuccessful outcome by age, sex, occupation, education, marital status, HIV risk group, residence in a rural district, adverse event during TB treatment, CTX use, prior history of TB, TB types, and registration status. In multivariate logistic regression analysis, we selected variables for inclusion based on plausibility, *a priori* evidence, completeness of data, or a p-value< = .20 in bivariate analysis. Variables were also checked for co-linearity and interactions and model fit assessed with the maximum likelihood test. Variables included in the final model included sex, the presence of an adverse event, rural residence, TB smear status and disease location (smear-positive vs. smear-negative vs. extrapulmonary), and CTX. We explored the robustness of our findings by repeating this analysis with all patients missing CTX data being re-defined as having received CTX and, separately, as having not received CTX. We also analyzed risk factors when we removed adverse event from the model and when we changed the outcome variable from the composite end-point of death, default, or failure to a composite of death and default or to just death.

### Ethical Review

The analyses reported in this manuscript were part of an evaluation of the TB/HIV program in An Giang province. CDC and the Vietnam National TB Program determined that this project represented a public health program evaluation, not human subjects research, and, therefore, did not require review by an institutional review board.

## Results

### Epidemiology of TB/HIV in An Giang

During the study period, An Giang province registered 21,964 TB patients, 637 (2.9%) of whom were HIV-infected. The number and proportion of HIV-infected TB patients increased over this period (139 [2.7%] in 2001, 150 [2.5%] in 2002, 161 [3.0%] in 2003, and 192 [3.5%] in 2004), but the increase was not statistically significant (p = 0.78 for trend). The incidence of HIV-associated TB per 100,000 persons was higher in urban areas (10–13 per 100,000) than rural areas (6–8 per 100,000) (p<.01). Observed incidence increased non-significantly in urban areas from 2001 (10 per 100,000) to 2003 (13 per 100,000) then declined slightly in 2004 (12 per 100,000). In rural areas, incidence was stable from 2001–2003 (6 per 100,000), then increased slightly in 2004 (8 per 100,000).

The median age of patients was 32 years (range 15–78). Most patients were male (79%), had low-income jobs or no jobs (60%), and had low education levels (76%).**[** **Table 1** **]** Of known HIV risk factors, 35% of patients reported that they had been clients of a commercial sex worker (CSW) and 11% were recorded as injection drug users. Among HIV-infected TB patients, 81% reported having at least one HIV-related condition at the time of HIV diagnosis.

**Table 1:** Demographic characteristics of HIV-infected TB patients (N = 637) – An Giang, Vietnam, 2001 – 2004.

Characteristic	Sub-category	n (%)
Sex	Male	501 (79)
	Female	136 (21)
Age	15–24	67 (11)
	25–34	321 (51)
	35–44	155 (24)
	45–54	71 (11)
	55 or older	21 (4)
	Missing	2 (<1)
Occupation	Farmer	107 (17)
	Worker (any type)	50 (8)
	Driver	36 (6)
	Unemployed	185 (29)
	Other (soldier, public servant and scholar)	140 (22)
	Missing	119 (19)
Education level	Illiterate	76 (12)
	Secondary	405 (64)
	High School	34 (5)
	College or University	2 (<1)
	Missing	120 (19)
HIV risk group	Sex with commercial sex worker	221 (35)
	Sex with stable partner	84 (13)
	Injection drug user	67 (11)
	Sex with multiple partner	34 (5)
	HIV-infected mother	11 (2)
	Men having sex with men	3 (<1)
	Missing	217 (34)
HIV symptoms at time of HIV diagnosis	Yes	514 (81)
	No	7 (1)
	Missing	116 (18)

**Figure 1:**
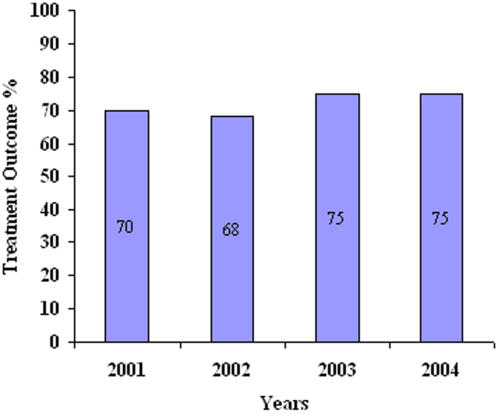
Trends in successful treatment outcomes for HIV-infected TB patients – An Giang, Vietnam, 2001–2004.

Seven percent of HIV-infected TB patients had previously been treated for TB. Smear-positive TB was diagnosed in 531 (83%), smear-negative TB in 29 (5%), and extra-pulmonary TB in 77 (12%).**[** **Table 2** **]** Successful treatment outcomes were persistently low, ranging from 68% to 75% for the years studied (p = 0.17 for trend).**[** **Figure 1** **]** For all years combined, 71% successfully completed treatment, 1% failed, 1% defaulted, 1% transferred out, and 26% died during TB treatment.

**Table 2:** Clinical characteristics of HIV-infected TB patients (n = 637) – An Giang, Vietnam, 2001 – 2004.

Characteristic	Sub-category	n (%)
History of TB	Yes	47 (7)
	No	582(93)
Smear/disease status	Pulmonary, smear-positive	531 (83)
	Pulmonary, smear-negative	29 (5)
	Extra-pulmonary	77 (12)
Registration status	New	587 (92)
	Previously treated	47 (7)
	Transfer in	3 (<1)
Symptoms at TB diagnosis	Cough^1^	605 (95)
	Fever^2^	447 (70)
	Weight loss^3^	347 (55)
Final TB treatment outcome	Cure	374 (59)
	Treatment completed	78 (12)
	Failure	6 (1)
	Died	167 (26)
	Default	9 (1)
	Transfer out	3 (1)

1 29 missing data

2 188 missing data

3 288 missing data

### Risk Factors for Unsuccessful Outcomes

In bivariate analysis, we found only one factor significantly associated (p<0.05) with an unsuccessful outcome: not being prescribed CTX. Of 453 patients prescribed CTX, 116 (26%) had an unsuccessful outcome compared with 33 (70%) of 47 who were not prescribed CTX (RR 0.4; CI, 0.3–0.5).**[** **Table 3** **]** Data about CTX use was missing for 133 patients; the relative risk of an unsuccessful outcome was similar in this group as those that were prescribed CTX. When restricting our analysis to the 47 patients not prescribed CTX, no factors were significantly associated with an unsuccessful outcome. Of all patients, 3 (75%) of 4 with an adverse event had an unsuccessful outcome compared with 179 (28%) of 630 without an adverse event (Relative Risk [RR] 2.6; 95% Confidence Interval [CI] 1.5–4.7; Fisher's exact p-value = 0.07).

**Table 3:** Bivariate analysis of risk factors for unsuccessful TB treatment outcomes among HIV-infected TB patients – An Giang, Vietnam, 2001 – 2004 (n = 634).*

Characteristic	Sub-category	Total	Unsuccessful outcome, n (%)	RR (95% CI)
Sex	Male	500	136 (27)	0.8 (0.6–1.0)
	Female	134	46 (34)	Ref
Age	15–24	67	18 (27)	0.9 (0.6–1.4)
	25–34	318	91 (29)	Ref.
	35–44	155	41 (26)	0.9 (0.7–1.3)
	45–54	71	25 (35)	1.4 (0.8–2.3)
	55 or older	21	6 (29)	1.0 (0.5–2.0)
	Missing	2	1 (50)	1.7 (0.4–7.1)
Education	Illiterate	76	22 (29)	1.0 (0.7–1.4)
	Secondary school	403	121 (30)	Ref.
	At least high school	36	9 (25)	0.8 (0.5–1.5)
	Missing	119	30 (25)	0.8 (0.6–1.2)
Marital status	Married	300	85 (28)	Ref.
	Single	143	45 (32)	1.1 (0.8–1.5)
	Divorced	32	9 (28)	1.0 (0.6–1.8)
	Separated	30	10 (33)	1.2 (0.7–2.0)
	Widowed	13	3 (23)	0.8 (0.3–2.2)
	Missing	116	30 (26)	0.9 (0.6–1.3)
HIV risk group	Men having sex with men	3	0	Undefined
	Sex with commercial sex worker	221	60 (27)	0.9 (0.5–1.6)
	Sex with stable partner	83	27 (323	1.2 (0.6–2.0)
	Sex with multiple partner	34	10 (29)	Ref.
	Injection drug user	67	25 (37)	1.3 (0.7–2.3)
	HIV-infected mother	11	2 (18)	0.6 (0.2–2.4)
	Missing	215	58 (27)	0.9 (0.5–1.6)
Residence	Urban area	165	52 (32)	Ref.
	Rural area	468	130 (28)	0.9 (0.7–1.2)
	Missing	1	0 (0)	Undefined
History of TB	Yes	47	11 (23)	0.8 (0.5–1.4)
	No	587	171 (29)	Ref.
Sputum/disease status	Pulmonary, smear-positive	528	155 (29)	Ref.
	Pulmonary, smear-negative	29	11 (38)	1.3 (0.8–2.1)
	Extra-pulmonary	77	16 (21)	0.7 (0.5–1.1)
Prescribed co-trimoxazole	Yes	454	116 (26)	0.4 (0.3–0.5)
	No	47	33 (70)	Ref.
	Missing	133	33 (25)	0.4 (0.3–0.5)
Adverse event during TB treatment	Yes	4	3 (75)	2.6 (1.5–4.7)†
	No	630	179 (28)	Ref.

* For this analysis, patients with an end-of-treatment outcome coded as “cure” or “completed” were characterized as having a “successful” outcome, and those coded as “failure” “default” or “died” as having an “unsuccessful” outcome. Patients with missing data were excluded from the analysis.

† Fisher's exact p-value = 0.07.

In multivariate analysis, the strongly protective effect of CTX persisted, after adjustment for sex, adverse event, rural residence, and TB smear status.**[** **Table 4** **]** The protective effect of CTX remained after re-defining all patients with missing CTX data as having received CTX and, separately, after re-defining all patients with missing CTX as having not received CTX. It also remained after we repeated all three of these analyses (primary analysis, CTX missing re-coded as received, CTX missing re-coded as not received) comparing patients with a successful outcome to only those that died or to those that died or defaulted, and after removing from the model the adverse event variable, which only occurred in a few patients.

**Table 4:** Multivariate analysis of risk factors for unsuccessful TB treatment outcomes among HIV-infected TB patients – An Giang, Vietnam, 2001 – 2004 (n = 633).

Characteristic	Sub-category	Adjusted OR (95% CI)	p-value
Adverse event during TB treatment		6.8 (0.7–71.5)	0.11
Residence in a rural area		1.0 (0.7–1.6)	0.87
TB smear status and location	Pulmonary, smear-positive	Ref	Ref
	Pulmonary, smear-negative	1.4 (0.6 – 3.1)	0.17
	Extra-pulmonary	0.6 (0.3–1.1)	0.05
Prescribed co-trimoxazole	Yes	0.1 (0.1–0.3)	<0.01
	No	Ref	Ref
	Missing	0.1 (0.1–0.3)	<0.01
Male		0.7 (0.4–1.0)	0.05

Of the 166 patients that died, the time between treatment initiation and death was known for 164 patients. The median time to death was 99 days (range 4 – 470 days) (mean 111.4 days). When stratified by CTX, the median time to death was 107 days in persons prescribed CTX, 74 days in persons not prescribed CTX, and 94 days in patients without known CTX status (p>.05 for all comparisons). Results were similar when comparing time to death or default (p>.05 for all comparisons).

## Discussion

Our retrospective review of routinely collected public health data from An Giang, Vietnam found that one in four persons with HIV-associated TB died during treatment, but TB treatment outcomes were significantly better in those taking CTX.

This is the first study to report an improvement in TB treatment outcomes among HIV-infected TB patients prescribed CTX living in Southeast Asia. Evidence for the benefit of CTX in HIV-infected TB patients comes from a randomized, clinical trial in the Ivory Coast, which demonstrated a 46% mortality reduction for HIV-infected TB patients taking CTX, and subsequent observational studies in Malawi and South Africa.[11]–[13] Despite these studies, a 2004 meta-analysis argued that the evidence in favor of treating all HIV-infected TB patients with CTX was limited and that further studies were needed.[14] Similarly, the WHO's Interim Policy on TB/HIV Collaborative Activities recommends CTX for HIV-infected TB patients living in Africa, but does not specify what should be done in other parts of the world, including Asia.[15] This study provides some support for applying the WHO recommendation about CTX to Vietnam.

The high mortality rate for HIV-infected TB patients in An Giang is consistent with data from other developing countries, but in marked contrast to the overall 92% success rate that the NTP reports nationally for treatment of smear-positive TB.[2] In fact, the mortality rate that we found in An Giang, and that reported from Ho Chi Minh City, is even higher than rates reported in sub-Saharan Africa.[4], [9] The high prevalence of HIV-related conditions reported by persons in this study suggests that late diagnosis of HIV may be one cause for the high mortality rate. Also, in sub-Saharan Africa, pulmonary, smear-negative TB is associated with higher mortality than other forms of diagnosed TB, but we did not find such an association in An Giang.[16] Further study is needed to understand whether these differences are real and, if they are, what their implications may be for responding to the TB/HIV syndemic in Vietnam.[17]

We found two unique features about the epidemiology of HIV-associated TB in An Giang. First, HIV-associated TB occurred most frequently in young men who reported sex with a commercial sex worker. The populations most at risk of acquiring HIV in Vietnam are IDU, commercial sex workers, and clients of commercial sex workers. Our review found that IDU comprised only a small proportion of HIV-infected TB patients in An Giang. Although this finding may be attributable to under-reporting, low rates of reported IDU are plausible, because this province is located far from large urban centers where injection drug use is more common.[18] Second, the vast majority of cases were smear-positive, which is uncommon in most HIV epidemic settings. Possible explanations include: insufficient case finding for TB in HIV-infected patients, in whom smear-negative and extra-pulmonary TB would be more common; a high prevalence of non-tuberculous mycobacteria; or inaccurate sputum microscopy. Given the high mortality rate, we think it is unlikely that the high proportion of smear-positive patients is attributable to patients having less advanced HIV disease.

This study has several major limitations. It was retrospective and based only on data that was available in clinical and public health records. We could not independently verify the accuracy of these records, nor could we collect additional data needed to confirm or refute our findings. This impacts variables such as those measuring the presence of an adverse event and CTX use. In particular, we cannot differentiate between patients prescribed CTX and patients who actually took CTX, nor can we know whether other unmeasured factors could account for differences in mortality rates between those taking CTX and those not taking it, including CD4 count, opportunistic infections, malaria, and diarrheal disease. The consistency and strength of association between CTX in our multivariate analysis, however, is reassuring.

An Giang has implemented the core activities recommended by WHO for responding to the TB/HIV syndemic, including HIV counseling and testing of TB patients, TB screening for HIV patients, and CTX preventive therapy. Nevertheless, the high mortality rate suggests that the quality and quantity of such activities may need to be increased and, even more important, that activities should be expanded to include ART, which has been demonstrated to be life-saving in HIV-infected TB patients and possibly INH preventive therapy, which has been shown to greatly reduce TB risk among HIV patients.[5], [6] This study indicates a major need to expand HIV-related care and treatment in An Giang and throughout Vietnam, and Vietnam has responded to this need by increasing HIV counseling and testing of TB patients, TB screening in HIV patients, and ART services. Evaluating data from these activities will help validate the findings from this study, identify other interventions effective at reducing mortality, and develop the critical evidence-base for responding to the TB/HIV syndemic.
